# 812. The Impact of Post-Operative Cephalexin on Surgical Site (SSI) Infections During a Cefazolin Shortage

**DOI:** 10.1093/ofid/ofab466.1008

**Published:** 2021-12-04

**Authors:** Sara Brown, R Brigg Turner, Dominic Chan

**Affiliations:** 1 Legacy Health, Portland, Oregon; 2 Pacific University, Hillsboro, Oregon

## Abstract

**Background:**

Drug shortages directly impact patient care. Rates of drug shortages have declined except for antimicrobials, where shortage rates remain similar each year.^1^ In November 2018, a national cefazolin shortage occurred driving health systems to implement a therapeutic interchange of cefazolin for cephalexin for post-operative antimicrobial prophylaxis. The objective of this study is to determine whether SSI-rates change when post-operative cephalexin is used in placed of cefazolin.

**Methods:**

This was a retrospective, observational cohort study of patients receiving post-operative antimicrobial prophylaxis at a community-based health system in Oregon and Washington between May 2018 – August 2019. Participants were divided into 3 periods for SSI-rate trend analysis: pre-shortage (May 2018 – October 2018), shortage (November 2018 – February 2019), and post-shortage (March 2019 – August 2019). The primary outcome was SSI-rates between groups.

**Results:**

There were 6,378 patients in total (5,840 cefazolin vs. 538 cephalexin). There were no significant differences in baseline characteristics of age, sex, body mass index (BMI), or hospital location. The rate of SSI between pre-shortage and post-shortage cefazolin groups was not statistically different (14 [0.5%] vs. 23 [0.8%]; p=0.16). The primary outcome of SSI in the shortage group who received cephalexin was not statistically different (37 [0.6%] vs. 0 [0%]; p=0.07).

**Conclusion:**

National drug shortages significantly impact patient care, often leading to seeking evidence-poor alternative medications. These results suggest cephalexin may be an acceptable post-operative prophylaxis antimicrobial if cefazolin is unavailable.

**Disclosures:**

**All Authors**: No reported disclosures

